# Does postoperative orbital volume predict postoperative globe malposition after blow-out fracture reconstruction? A 6-month clinical follow-up study

**DOI:** 10.1007/s10006-019-00748-3

**Published:** 2019-02-12

**Authors:** Johanna Snäll, M. Narjus-Sterba, M. Toivari, T. Wilkman, H. Thorén

**Affiliations:** 1 Department of Oral and Maxillofacial Diseases, University of Helsinki and Helsinki University Hospital, Helsinki, Finland; 2 Department of Oral and Maxillofacial Diseases, University of Turku and Turku University Hospital, Turku, Finland

**Keywords:** Orbital fracture, Blow-out fracture, Globe malposition

## Abstract

**Purpose:**

The aim of this study was to investigate the relationship between intraorbital volume change caused by orbital fracture and globe malposition (GMP) in blow-out fracture patients undergoing surgery and to clarify the significance of different radiologically detected predictors associated with GMP.

**Patients and methods:**

A 6-month prospective follow-up study of unilateral isolated orbital fractures was designed and implemented. The main outcome variable was GMP (present or absent); the secondary outcome was orientation of GMP (horizontal or vertical). The primary predictor variable was postoperative orbital volume difference determined as the difference between the fractured and non-fractured orbit (measured in milliliter and analyzed in milliliter and percentages). The explanatory variables were gender, age, treatment delay from trauma to surgery, fracture site, horizontal depth of the fracture, fracture area, maximum vertical dislocation of the fracture, and preoperative volume difference.

**Results:**

A total of 15 patients fulfilled the inclusion criteria and were followed for 6 months from a larger cohort. GMP was detected in 6/15 patients (40.0%). GMP was more often present in large (≥ 2.5 cm^2^) fractures (55.6%), in combined orbital fractures (50.0%), and in fractures with preoperative volume difference ≥ 2.5 ml (62.5%) regardless of the postoperative volume correction. Postoperatively, patients with and without GMP displayed overcorrection of orbital volume; 4.15% corresponded to 1.15 ml (with GMP) and 7.6% corresponded to 1.9 ml (without GMP).

**Conclusion:**

GMP was present in large and combined orbital fractures. Clinically detectable postoperative GMP occurred despite satisfactory orbital reconstruction and overcorrection. Mild GMP, however, is not significant for the patient.

## Introduction

Orbital fracture patients may have globe malposition (GMP) due to posttraumatic and postoperative changes in bony and soft tissue support. In addition to cosmetic sequelae of the globe position itself, malposition may also lead to other sequelae such as differences in interpalpebral width [1] and may occur simultaneously with diplopia [2, 3].

Due to soft tissue swelling, the natural globe position often cannot be evaluated at the beginning of follow up. In addition, the final assessment should be made after final tissue healing; thus, conclusions with long-term significance are required. A correlation between GMP and volume change has been demonstrated [4–6]. Considering individual differences in orbit volume, an increase of 10% to 15% has been shown to cause 1.5- to 2-mm enophtalmos [7]. Inadequate fracture reduction and incomplete surgical reconstruction have been described as the main reasons for postoperative enophtalmos [6, 8].

In addition to volume increase, the role of soft tissue prolapse and soft tissue damage in GMP has been elucidated. Orbital fat scar formation in the retrobulbar space and fat atrophy has been described [5, 9–11]. Furthermore, soft tissue changes and size of the primary fracture area have been shown to predict postoperative enophtalmos independent of surgery [12]. Thus, the clinical significance of postoperative volume change should be analyzed in more detail and compared with other fracture-related factors.

The primary aim of the present study was to clarify the significance of postoperative orbital volume in postoperative long-term GMP in isolated blow-out fracture patients. The secondary aim was to evaluate whether there is an association between fracture size, type, and location to postoperative GMP. We hypothesized that postoperative GMP is predictable from measurements based on preoperative and postoperative computer tomography images.

## Patients and methods

### Study design and inclusion criteria

This study was part of a larger cohort of facial fracture patients of at least 18 years of age who participated in a prospective study of patients’ quality of life after surgery between the years 2006 to 2010 in the Department of Oral and Maxillofacial Surgery, Helsinki University Hospital. The present study included patients with an isolated, unilateral orbital fracture reconstructed with a titanium mesh. Patients with orbital fracture extending to the orbital rim, those requiring surgery for other facial fractures, or both were excluded.

A prospective clinical follow-up of 6 months was required for the final analyses of the present study. Multi-slice computed tomography (MSCT) scanners (GE Healthcare, Milwaukee, WI) with a bone algorithm were used for computer tomography imaging in all participants preoperatively and immediately postoperatively. The data was reformatted into 1.0- or 1.5-mm-thick coronal, axial, and sagittal images. The orbital volumes and radiological measurements were analyzed retrospectively.

### Study variables

The primary outcome variable was presence of GMP, defined as ≥ 2-mm difference compared to the unaffected eye. The secondary outcome variable was direction of GMP divided in enophtalmos (horizontal GMP) and hypophtalmos (vertical GMP).

The primary predictor variable was postoperative orbital volume difference (measured in milliliter and analyzed in milliliter and percentages). Volume difference was determined as the difference between the fractured and non-fractured orbit.

The explanatory variables were gender, age (< 50 or ≥ 50 years), treatment delay from trauma to surgery (< 6 or ≥ 6 days), fracture site (patients with an orbital floor fracture or patients with a combined orbital floor and medial wall fracture), horizontal depth of the fracture (posterior orbital third fractured or non-fractured), fracture area (small, dislocated fracture area < 2.5 cm^2^; large, dislocated fracture area ≥ 2.5 cm^2^), maximum dislocation of the fracture (< 10 or ≥ 10 mm), and preoperative orbital volume difference.

### Clinical examinations

GMP was measured clinically by one examiner (J.S.) at 6 months postoperatively. Examination of enophtalmos was performed with a Keeler® exoftalmometer. Hypophtalmos was measured using optician’s examination spectacles provided with a vertical milliliter scale. Two consecutive measurements of the same value were required for the recorded value. GMP was defined as a difference ≥ 2 mm between the fractured and non-fractured orbit.

### Radiological analyses

Radiologic analysis for the area of the dislocated fracture, depth of the fracture, and maximum dislocation of the fracture was performed twice by an experienced maxillofacial radiologist to secure the measurements by intrainvestigator correlation. Depth of the fracture in anteroposterior direction was based on orbital zone classification presented by Jaquiéry et al. [13]. The posterior orbital third was recorded as fractured or non-fractured.

### Volume measurements

Preoperative and postoperative measurements of orbital volume were compared with the non-fractured orbit. The one-click method defines the orbital volume by marking the outer orifice of the optic canal in the apex of the orbit. The accurate location of this point is further adjusted with an algorithm using the predefined form of the canal (Wilkman et al., submitted). The orbital volume embraces the outer orifice of the optic canal in the apex and the orbital rim anteriorly. The orbital volumes were analyzed based directly on DICOM data using Disior Ltd. proprietary algorithms and solved numerically.

A volume difference variation < 1.0 ml was the accepted measurement accuracy in non-fractured orbits in preoperative and postoperative images.

## Results

Of the 27 patients with unilateral orbital fracture recruited for the initial study, 2 patients refused to participate. Of the remaining 25 patients, 19 fulfilled the inclusion criteria of orbital reconstruction with titanium mesh. Of these 19 patients, 4 were lost to follow-up and were excluded. Thus, a total of 15 patients were included in the final analyses. A 6-month follow-up was performed between days 158 to 234 (median 188 days).

Descriptive statistics of the patients are shown in Table 1. Nine patients were female (60.0%). Age ranged from 22.5 to 81.1 years (median 50.0 years). Time span from initial trauma to surgery ranged from 2 to 19 days (median 5 days). The orbital floor was fractured exclusively in seven patients (46.7%); the remaining eight patients had a combined fracture of orbital floor and medial wall. Five patients (33.3%) had orbital posterior third fractured. Fracture area was large (≥ 2.5 cm^2^) in nine patients (60.0%). Maximum fracture depth varied from 3 to 33 mm; eight patients (53.3%) had a fracture depth ≥ 10 mm. Orbital volume difference ranged preoperatively from − 1.6 to 6.3 ml (median 2.5 ml) (− 6.2% to 24.8%, median − 8.0%). Postoperative volume difference ranged from − 2.7 to 0.9 ml (median − 1.7 ml) (− 11.7% to 3.7%, median − 6.3%). GMP was detected in six patients. Three patients had enophtalmos and two had hypophtalmos. One patient had both enophtalmos and hypophtalmos.

**Table 1:** Descriptive statistics of 15 patients with an isolated orbital blow-out fracture

	No. of patients	% of *n* = 15
Gender		
Male	6	40.0
Female	9	60.0
Age		
Range	22.5 to 81.1	
Median	50.0	
Treatment delay (days)		
Range	2 to 19	
Median	5	
Fracture site		
Isolated floor	7	46.7
Combined orbital floor and medial wall	8	53.3
Posterior orbital third fractured		
Yes	5	33.3
No	10	66.7
Fracture area		
Small (< 2.5 cm^2^)	6	40.0
Large (≥ 2.5 cm^2^)	9	60.0
Maximum fracture depth (mm)		
Range	3 to 33	
Median	10	
Preoperative orbital volume difference*		
Milliliters		
Range	− 1.6 to 6.3	
Median	2.5	
Percentages		
Range	− 6.2 to 24.8	
Median	8.0	
Postoperative volume difference*		
Milliliters		
Range	− 2.7 to 0.9	
Median	− 1.7	
Percentages		
Range	− 11.7 to 3.7	
Median	− 6.3	
Globe malposition ≥ 2 mm at 6 months		
Yes	6	40.0
No	9	60.0
Enophtalmos		
Yes	4	26.7
No	11	73.3
Hypohtalmos		
Yes	3	20.0
No	12	80.0

*Volume difference compared with non-fractured orbit

Associations between GMP and the primary predictor and explanatory variables are shown in Table 2. Associations with direction of GMP divided in enophtalmos (horizontal GMP) and hypophtalmos (vertical GMP) are shown in Table 3. Patients with a fracture area ≥ 2.5 cm^2^ had GMP more often than patients with smaller fractures (55.6% versus 16.7%). All four patients with enophtalmos had combined medial wall and floor fracture. None of these patients had posterior third fracture. Preoperative orbital volume was ≥ 2.5 ml in 5/6 patients with GMP. Statistical differences could not be calculated due to the small number of patients.

**Table 2:** Association between presence of GMP and orbital volume changes and explanatory variables at 6 months postoperatively

	GMP present		GMP absent	
	*n*	% of *n*	*n*	% of *n*
Gender				
Male (*n* = 6)	3	50.0	3	50.0
Female (*n* = 9)	3	33.3	6	66.7
Age group				
< 50 years (*n* = 7)	2	28.6	5	71.4
≥ 50 years (*n* = 8)	4	50.0	4	50.0
Treatment delay				
< 6 days (*n* = 8)	4	50.0	4	50.0
≥ 6 days (*n* = 7)	2	28.6	5	71.4
Fracture site				
Orbital floor (*n* = 7)	2	28.6	5	71.4
Combined orbital floor and medial wall (*n* = 8)	4	50.0	4	50.0
Posterior orbital third fractured				
Yes (*n* = 5)	2	40.0	3	60.0
No (*n* = 10)	4	40.0	6	60.0
Fracture area				
Small (< 2.5 cm^2^) (*n* = 6)	1	16.7	5	83.3
Large (≥ 2.5 cm^2^) (*n* = 9)	5	55.6	4	44.4
Maximum fracture depth				
< 10 mm (*n* = 7)	3	42.9	4	57.1
≥ 10 mm (*n* = 8)	3	37.5	5	62.5
Preoperative orbital volume difference*				
Milliliters				
< 2.5 ml (*n* = 7)	1	14.3	6	85.7
≥ 2.5 ml (*n* = 8)	5	62.5	3	37.5
Percentages				
< 8.0% (*n* = 7)	2	28.6	5	71.4
≥ 8.0% (*n* = 8)	4	50.0	4	50.0
Postoperative orbital volume difference*				
Milliliters				
< − 1.7 ml (*n* = 7)	2	28.6	5	71.4
≥ − 1.7 ml (*n* = 8)	4	50.0	4	50.0
Percentages				
< − 6.3% (*n* = 7)	2	28.6	5	71.4
≥ − 6.3% (*n* = 8)	4	50.0	4	50.0

*Volume difference compared with non-fractured orbit

**Table 3:** Association between direction of GMP and orbital volume changes and explanatory variables at 6 months postoperatively

	Direction of GMP			
	Enophtalmos present		Hypophtalmos present	
	*n*	% of *n*	*n*	% of *n*
Gender				
Male (*n* = 6)	3	50.0	1	16.7
Female (*n* = 9)	1	11.1	2	22.2
Age group				
< 50 years (*n* = 7)	2	28.6	0	
≥ 50 years (*n* = 8)	2	25.0	3	37.5
Treatment delay				
< 6 days (*n* = 8)	3	37.5	2	25.0
≥ 6 days (*n* = 7)	1	14.3	1	14.3
Fracture site				
Orbital floor (*n* = 7)	0	0	2	28.6
Combined orbital floor and medial wall (*n* = 8)	4	50.0	1	12.5
Posterior orbital third fractured				
Yes (*n* = 5)	0	0	2	40.0
No (*n* = 10)	4	40.0	1	10.0
Fracture area				
Small (< 2.5 cm^2^) (*n* = 6)	1	16.7	0	0
Large (≥ 2.5 cm^2^) (*n* = 9)	3	33.3	3	33.3
Maximum fracture depth				
< 10 mm (*n* = 7)	3	42.9	1	14.3
≥ 10 mm (*n* = 8)	1	12.5	2	25.0
Preoperative orbital volume difference*				
Milliliters				
< 2.5 ml (*n* = 7)	0	0	1	14.3
≥ 2.5 ml (*n* = 8)	4	50.0	2	25.0
Percentages				
< 8.0% (*n* = 7)	1	14.3	2	28.6
≥ 8.0% (*n* = 8)	3	37.5	1	12.5
Postoperative orbital volume difference*				
Milliliters				
< − 1.7 ml (*n* = 7)	2	28.6	0	0
≥ − 1.7 ml (*n* = 8)	2	25.0	3	37.5
Percentages				
< − 6.3% (*n* = 7)	2	28.6	0	
≥ − 6.3% (*n* = 8)	2	25.0	3	37.5

*Volume difference compared with non-fractured orbit

*GMP* globe malposition

Clinical findings, postoperative volumes, and radiological measurements of 15 patients and differences between patients with and without GMP are presented in Table 4. Preoperative orbital volume was slightly greater in patients with postoperative GMP than in patients without (12.6%, 3.1 ml versus 4.6%, 1.4 ml). GMP occurred despite sufficient surgical reconstruction and volume overcorrection. Measurements showed volume overcorrection in patients with (4.15% corresponding to 1.15 ml) and without GMP (7.6% corresponding to 1.9 ml).

**Table 4:** Clinical findings and volume and fracture measurements in 15 blow-out fracture patients

	Globe malposition			Preoperative volume difference		Postoperative volume difference		Fracture site**	Posterior orbital third fractured	Fracture area***	Maximum dislocation of the fracture (mm)
	Any	Enophtalmos (mm)	Hypophtalmos (mm)	ml*	%*	ml*	%*				
1	Yes	− 2		5.1	17.9	− 2.2	− 8.0	Combined		Large	10
2	Yes	− 2	− 2	2.5	7.7	− 0.6	− 2.0	Combined		Large	8
3	Yes	− 2		3.3	14.3	− 2.7	− 11.7	Combined		Large	8
4	Yes		− 2	6.3	24.8	0.9	3.7	Floor	Yes	Large	12
5	Yes		− 4	0.6	2.4	− 1.7	− 6.3	Floor	Yes	Large	12
6	Yes	− 2		2.9	10.9	0.3	1.1	Combined		Small	3
Median				3.1	12.6	− 1.15	− 4.15				9
7	No			1.4	4.5	− 1.2	− 4.1	Combined		Small	4
8	No			− 0.2	− 0.8	− 2.5	− 8.6	Combined	Yes	Large	8
9	No			− 1.6	− 6.2	− 2.3	− 9.3	Floor		Large	6
10	No			2.7	8.0	− 1.9	− 5.8	Floor		Large	10
11	No			1.4	4.6	− 2.7	− 8.4	Floor	Yes	Small	8
12	No			4.1	12.6	− 2.4	− 7.6	Combined		Large	10
13	No			2.0	9.5	− 1.6	− 8.1	Floor	Yes	Small	10
14	No			4.1	15.8	0.7	2.6	Combined		Small	12
15	No			0.6	2.9	0	0	Floor		Small	33
Median				1.4	4.6	− 1.9	− 7.6				10

***Volume difference compared with non-fractured orbit; combined = medial wall and floor; ***dislocated fracture area was classified as small (< 2.5 cm^2^) and large (≥ 2.5 cm^2^)

## Discussion

**Fig. 1:**
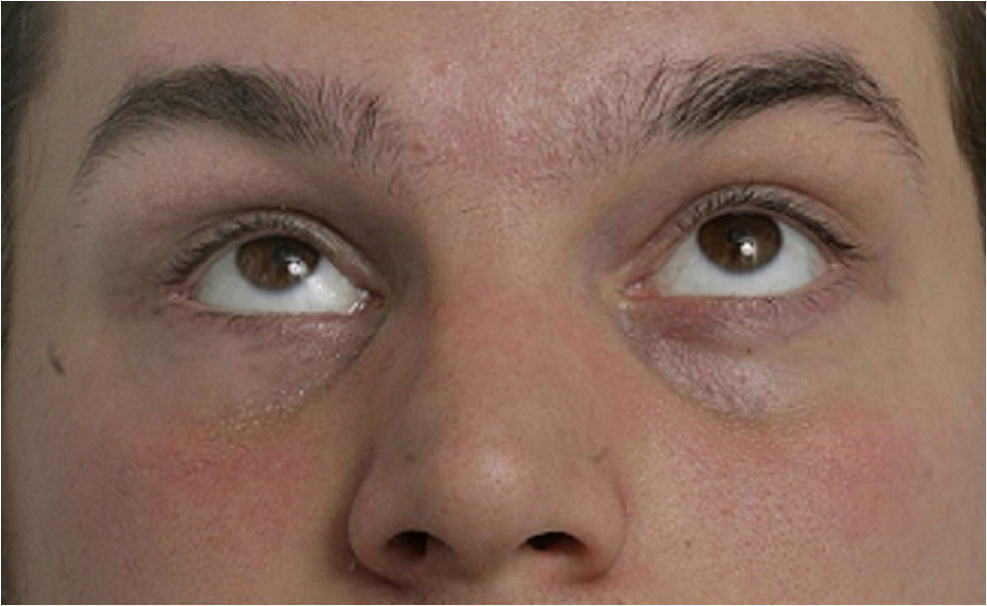
This patient with a large combined orbital floor and medial wall fracture had − 2-mm enophtalmos at 6 months postoperatively. Globe malposition occurred despite notable overcorrection of the bony orbit (11.7%, 2.7 ml)

The present study aimed to clarify the significance of postoperative orbital volume in postoperative long-term GMP. We also wanted to study the association between fracture size, type, and location to GMP. We hypothesized that GMP can be predicted from these measurements. Our hypothesis was partially realized. A trend towards postoperative GMP in wide fractures was detected. However, despite acceptable volume restoration in all remaining patients, six patients had GMP (Fig. 1).

A previous prospective multicenter study of 195 orbital fracture patients showed significantly better volume correction with individualized implants compared with non-customized implants [14]. Interestingly, a more precise volume correction in patients with individualized implants showed no difference in clinical outcome between the study groups. Oh et al. [15] also showed that the clinical long-term difference may be negligible even if the postoperative orbital volume remains greater than that of the intact orbit. In the present study, increased volumes were successfully corrected but GMP still occurred. The maximum postoperative volume increase was not greater than 0.9 ml (3.7%). Overcorrection up to 2.7 ml (11.7%) also did not prevent postoperative GMP. These results emphasize that postoperative volume measurements have a surprisingly minor impact on long-term clinical outcome.

The fractured area is an important predictor for enophtalmos even in patients undergoing surgery [12]. It has been reported to be a more important predictor for GMP than preoperative enophtalmos [4, 16]. In addition, GMP has been shown to be more severe in patients with combined floor and medial wall fracture than with pure floor fractures [17]. In the present study, 5/6 patients with postoperative GMP had fracture with dislocated area > 2.5 cm^2^, and all four patients with enophtalmos had a combined fracture of medial wall and floor. Patients with GMP also had greater preoperative orbital volume. It can be assumed that restoring the volume in wide fractures is not sufficient to achieve a precise clinical outcome.

The fracture extent has a positive correlation with soft tissue impairment. Kim et al. [11] emphasized the significance of preoperatively herniated soft tissue in late enophtalmos instead of fracture size. In addition, other studies [5, 9, 10] have described orbital fat scarring and atrophy as a source for enophtalmos. Contrasting results on the relationship between fat atrophy and enophtalmos have also been presented. Ramieri et al. [8] demonstrated that enophtalmos correlates with orbital volume and height of the retrobulbar portion of the orbit as opposed to fat atrophy. In isolated orbital blow-out fractures, where the energy is transmitted directly through blunt impact to the orbital internal structures [18], periosteal and orbital soft tissue typically explode outside the orbit. The mechanism of blow-out fractures differs from other orbital fracture types. Preservation or breakdown of periosteal support and size of the prolapsed soft tissue should be considered in further GMP studies. Thus far, postoperative orbital tissue remodeling is not well predictable [19].

**Fig. 2.**
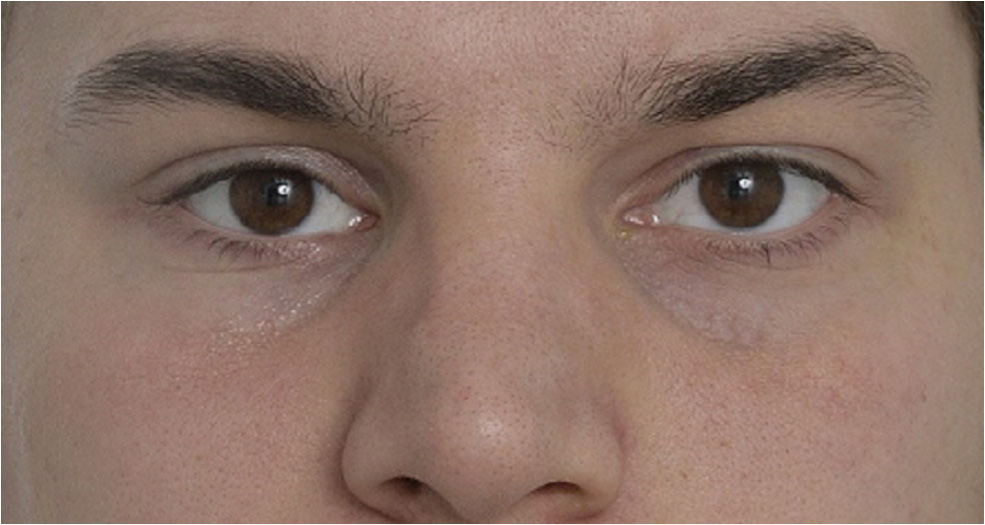
The same patient (Fig. 1) was satisfied with the final outcome a year after surgery despite mild globe malposition (− 2-mm enophtalmos and − 1-mm hypophtalmos)

Clinically, detectable GMP differs from GMP that has subjective importance for the patient. Yong et al. demonstrated that enophtalmos of 2.1 mm is subjectively insignificant to patients [20]. In our study, GMP of ≥ 2 mm was measured in 6/15 patients. The widest difference was in a patient with hypophtalmos of 4 mm. None of these patients suffered from double vision that interfered with daily activities. All patients with GMP were satisfied with the outcome and none experienced GMP such that repair was desired (Fig. 2). According to our study, patients are satisfied with the final outcome if the orbital volume is restored with a slight overcorrection (1.7 ml, 6.3% in median).

The finding that observable postoperative GMP occurs despite satisfactory surgery poses the question of whether surgical restoration is necessary in patients with minor symptoms or findings. Bruneau et al. [12] showed in their study of 34 isolated orbital floor fractures that GMP and diplopia were not resolved with surgery. The final clinical outcome was not better in patients with surgery compared with patients who did not undergo surgery. Similarly, Alinasab et al. [21] presented long-term results of orbital floor fracture patients who were not treated surgically and showed no correlation between large changes of orbital volume and GMP. Yong et al. [20] showed significant soft tissue and bone remodeling in blow-out fracture patients who did not undergo surgery. Somewhat surprisingly, this study showed orbital volume decrease without surgery during long-term follow up. These findings indicate that there are several other factors in addition to orbital volume which should be evaluated when achieving optimal clinical outcome.

Our volume analysis is based on DICOM data, where the missing data between imaging slices cause uncertainty in analysis due to discontinuous orbital form. However, the difference between non-fractured orbits was < 1.0 ml, which was sufficient in the present study. Comparable accuracy was reported in a previous study of computer-aided orbital volume measurements based on 3D-shape analysis, where the interobserver and intraobserver variability was shown to be accurate down to 1.0 ml [22]. A milliliter difference should be considered minor. A mean difference between intact human right and left orbital volume was shown to be 0.44 ml [23]. Imaging accuracy and technical development will surely provide more detailed volume measurements in the future.

Our observations indicate that follow-up studies are required to clarify the long-term outcome in orbital fracture patients. In addition to fracture size and location, additional imaging at a later stage would provide evidence of clinical significance of periorbital soft tissue injury as a GMP predictor.

## Conclusion

In the present study, GMP occurred despite satisfactory volume reconstruction. The primary extent of the fracture is an important predictor when considering long-term postoperative outcomes.
